# Physical Properties of Schwarzschild–deSitter Event Horizon Induced by Stochastic Quantum Gravity

**DOI:** 10.3390/e23050511

**Published:** 2021-04-23

**Authors:** Claudio Cremaschini, Massimo Tessarotto

**Affiliations:** 1 Research Center for Theoretical Physics and Astrophysics, Institute of Physics, Silesian University in Opava, Bezručovo nám.13, 746 01 Opava, Czech Republic; maxtextss@gmail.com; 2 Department of Mathematics and Geosciences, University of Trieste, Via Valerio 12, 34127 Trieste, Italy

**Keywords:** covariant quantum gravity, cosmological constant, Schwarzschild–deSitter space-time, event horizon, stochastic effects, tunneling phenomena, 03.50.-z, 03.65.-w, 04.20.-q, 04.20.Cv, 04.60.-m, 04.60.Bc, 04.70.Dy

## Abstract

A new type of quantum correction to the structure of classical black holes is investigated. This concerns the physics of event horizons induced by the occurrence of stochastic quantum gravitational fields. The theoretical framework is provided by the theory of manifestly covariant quantum gravity and the related prediction of an exclusively quantum-produced stochastic cosmological constant. The specific example case of the Schwarzschild–deSitter geometry is looked at, analyzing the consequent stochastic modifications of the Einstein field equations. It is proved that, in such a setting, the black hole event horizon no longer identifies a classical (i.e., deterministic) two-dimensional surface. On the contrary, it acquires a quantum stochastic character, giving rise to a frame-dependent transition region of radial width δr between internal and external subdomains. It is found that: (a) the radial size of the stochastic region depends parametrically on the central mass *M* of the black hole, scaling as $\delta r∼M^{3}$; (b) for supermassive black holes δr is typically orders of magnitude larger than the Planck length $l_{P}$. Instead, for typical stellar-mass black holes, δr may drop well below $l_{P}$. The outcome provides new insight into the quantum properties of black holes, with implications for the physics of quantum tunneling phenomena expected to arise across stochastic event horizons.

## 1. Introduction

This paper is part of the research effort devoted to the quantum regularization of singular classical black hole (BH) solutions. In fact, an ubiquitous property of classical General Relativity (GR) is related to the occurrence of coordinate singularities in the line-element representation of the space–time metric tensor solution of the Einstein field equations (EFE). In the case of black hole geometry, this feature can identify either the singularity at the black hole center (the so-called essential singularity) or the one at the event horizon (EH) [1,2,3]. At present, the prevailing opinion is that space–time singularities should be regarded as signatures of possible quantum effects occurring in the presence of intense gravitational fields. This is indeed one of the main motivations that lies behind the investigation of strong field regimes of gravity through the direct observation and detection of gravitational waves and BHs. Such an occurrence is interpreted at the same time as the manifestation of the limits of the theory of classical GR and its description of gravitational field. This, ultimately, shows the necessity of recurring to a quantum gravity theory for its consistent resolution [4,5,6,7].

A recent advance in this direction is the study proposed in reference [8]. Based on the theory of manifestly covariant quantum gravity (CQG-theory, see [9,10,11]), it has been proved that, provided a non-vanishing quantum cosmological constant (CC) is present, a regular background space–time metric tensor can effectively be obtained starting from a singular one. This is reached by constructing suitable scale-transformed and conformal solutions for the metric tensor. Accordingly, the conformal scale form factor is determined by the quantum Hamilton equations underlying the quantum gravitational field dynamics of CQG-theory.

However, in all black hole geometries, another kind of singular behavior actually occurs at event horizons. Its origin is related to the very nature of event horizons in GR: they are, in fact, deterministic two-dimensional surfaces which divide space–time into mutually “incommunicable” (or impenetrable) subdomains. This is a very unnatural behavior in quantum mechanics, where absolute barriers cannot exist. For this reason, EHs might/should be regarded as a natural candidate for a variety of possible quantum effects. The purpose of this investigation is to address one of them, related to the recent discovery of the stochastic nature of the cosmological constant.

The nature of all EH singularities in classical GR is straightforward. For definiteness, let us consider a space–time represented by the differential manifold $\left\{Q^{4},\hat{g}\left(r\right)\right\}$ in which $Q^{4}$ is a time-oriented, 4−dimensional, Riemannian spacetime with signature $\left\{1,-1,-1,-1\right\}$. Here, $\hat{g}\left(r\right)$ denotes the “background” classical metric field tensor, which is assumed to be smoothly parametrized with respect to the coordinate system $r\equiv \left\{r^{\mu }\right\}$ and is defined via its covariant and countervariant coordinate representations $\left\{{\hat{g}}_{\mu \nu }\left(r\right)\right\}$ and $\left\{{\hat{g}}^{\mu \nu }\left(r\right)\right\}$. Then, the equation

(1) A(r)=0

prescribes an event horizon, with A(r) denoting the Riemannian quadratic form $A\left(r\right)\equiv {\hat{g}}_{\mu \nu }\left(r\right)dr^{\mu }dr^{\nu }$. It may happen, accidentally, that some of the components of ${\hat{g}}_{\mu \nu }\left(r\right)$ diverge, thus giving rise to an apparent singularity. However, such occurrences can always be ruled out (i.e., “regularized”) by means of suitable changes in GR-frame (coordinate system), i.e., local point transformations (LPT) of the type

(2) $$r\to r^{\prime }=r^{\prime }\left(r\right),$$

which leave the differential manifold of spacetime $\left\{Q^{4},\hat{g}\left(r\right)\right\}$ unchanged. This happens because they arise solely from the choice of coordinates and disappear by a suitable re-definition of the coordinate system. In fact, the change in the signature of the quadratic form A(r) can always be realized in such a way that the tensor ${\hat{g}}_{\mu \nu }\left(r\right)$ remains finite. As a consequence, EH singularities are commonly referred to as coordinate or apparent singularities.

On the other hand, in classical GR, the background metric tensor $\hat{g}\left(r\right)$ is identified with a solution to the Einstein field equations and, as such, it is deterministic. This conclusion implies that Equation (1), i.e., the event horizon itself, necessarily identifies a true physical space–time frontier with a well-defined deterministic character. Such a feature can be viewed, in fact, as representative of a kind of singular behavior. This is because, in classical GR, EHs effectively separate two neighboring, i.e., infinitely close but absolutely “incommunicable”, regions of space–time with different metric signatures (i.e., respectively, A(r)<0 and A(r)>0). The question which arises is, therefore, whether the concept of deterministic EH is at all compatible with quantum gravity.

It must be stressed that the very notion of EH has gained great attention in recent decades among the scientific community (of theoretical and mathematical physicists in particular). Indeed, challenging questions are several. These concern, for example, the nature and existence of EHs, their mathematical description, the investigation of quantum and thermodynamics phenomena that can occur in their surroundings, the interaction with particles and fields, and the role of the gravitational field itself [12,13,14]. Finally, it is well known that event horizons may be characterized by the occurrence of Hawking radiation [15,16,17], as well as energetic particle phenomena and collective interactions [18,19,20,21]. For this reason, they are also expected to provide the background for quantum field phenomena, like tunneling effects [22,23]. These may ultimately involve the dynamics of the same gravitational field [24,25], the physics of event horizons [26], as exemplified by particle emission and acceleration mechanisms [27], and phenomena related to entropy creation/conservation [28,29,30].

However, the debate on whether physical event horizons, i.e., those occurring in the presence of quantum gravity effects, are really absolutely impenetrable (domains) or not remains controversial. The task of this paper is to address such an issue.

A satisfactory, physically grounded answer could have relevant implications, both for cosmology and field theories in general. The issue is intriguing because, ultimately, it is about the fundamental question of “communication” across EHs, i.e., the possibility of the transition of information and matter (either classical or quantum) across the same space–time surfaces. On the other hand, from the quantum viewpoint, the very notion of impenetrable surfaces like the EH appears intrinsically unphysical. This suggests that, ultimately, EHs should manifest a quantum stochastic nature of some sort, possibly arising in localized subdomains characterized by prescribed space, time and energy scales. As recently discovered in reference [31], an instance of this type—which pertains the treatment of the cosmological deSitter EH—is expected to occur in the framework of the manifestly covariant theory of quantum gravity (CQG-theory). This refers to the crucial discovery of the stochastic character of the cosmological constant, which, in the context of CQG-theory, is produced by the non-linear graviton vacuum–Bohm interaction [32,33,34]. The conclusion is determined by the stochastic character of the related self-consistent quantum PDF. This yields corresponding stochastic quantum-modified Einstein field equations, which were shown to admit a stochastic cosmological deSitter solution for the space–time metric tensor. As a consequence, it was proved that, in such a setting, the location of the quantum-modified deSitter EH becomes stochastic too. As a physical application, the expression of the Hawking temperature defined on such a surface was carried out. This proved that the stochastic behavior of quantum gravity can affect the thermodynamic description of continuum gravitational field and the related particle-tunnelling effect that might arise across the stochastic horizon boundary in a non-trivial way.

These premises suggest, at least, the obvious possibility that the radius of black hole event horizons should not be regarded as a deterministic quantity. Rather, it should be treated as having some sort of stochastic character. Such effects, it is understood, should also occur when appropriately small scales are considered (e.g., in particular, a suitably small neighborhood of the EH is considered). However, what these characteristic lengths should be remains essentially unknown because of the lack of a reliable quantum physical model. In this regard, it should be stressed that the Planck length might not be relevant at this stage.

The basic consequence is, therefore, that regions with different metric signatures should not be regarded as impenetrable, both for classical matter and radiation, and for quantum particles and fields. Therefore, the conjecture arises whether stochastic, i.e., quantum effects, in the prescription of an (otherwise classical) event-horizon surface might locally arise. This could give rise to a new kind of quantum tunnelling effect. In fact, in such a case, a particle might have a finite probability density of simultanrously being either “in” or “out” with respect to a stochastic surface which is no-longer prescribed as a deterministic barrier.

Given these premises, this paper is intended to be a continuation of previous research effort dealing with the investigation of the quantum modifications/corrections to the structure of classical black holes and the physics of event horizons in the presence of stochastic gravitational fields. The theoretical framework of reference is identified again with the manifestly covariant theory of quantum gravity (CQG-theory) and the prescription of a stochastic quantum PDF. The latter is associated with a stochastic cosmological constant produced by the non-linear quantum-vacuum Bohm interaction among massive gravitons, and is characteristic of CQG-theory. For this purpose, the specific case of the Schwarzschild–deSitter geometry is considered, and the consequent stochastic modifications of the Einstein field equations and of its corresponding background space–time metric tensor are investigated. The basic goals are as follows:

- To show that CQG-theory modifies the classical solution of the Schwarzschild–deSitter configuration, characterized by spherical symmetry and generated by a central mass *M* and the coexistence of a stochastic quantum-generated cosmological constant Λ. In such a setting, the black hole (inner) EH is no longer identified with a classical two-dimensional surface separating internal and external subdomains. On the contrary, it acquires a quantum stochastic character, giving rise to a transition region of radial width δr between the same subdomains. Such a subdomain is referred to here as *stochastic EH belt*;
- To show that, when measured in a co-moving frame originating in the center of the BH, the typical radial size of the stochastic EH belt (δr) is not a universal constant or a parameter-independent quantity. More precisely, the frame-dependent quantity δr is shown to depend parametrically on the central mass *M* of the black hole and, in particular, to scale as $\delta r∼M^{3}$. We intend to show that δr differs generally from the Planck length $l_{P}$, which is an invariant length customarily claimed as the characteristic size for the manifestation of black-hole quantum-gravity phenomena;
- To display explicit numerical estimates of the radial width δr in sample cases, which demonstrate the occurrence of such a feature. These show, in particular, that in the case of supermassive black holes with mass in the range $M\in \left[10^{6}-10^{10}\right]M_{⊙}$, δr are typically orders of magnitude larger that the Planck length $l_{P}$. Then, $l_{p}≲\delta r\ll l_{p}$ for intermediate-mass black holes with mass in the range $M\in \left[10^{2}-10^{4}\right]M_{⊙}$. Instead, for typical stellar-mass black holes with mass $M∼10M_{⊙}$, δr may drop well below $l_{P}$;
- To ascertain the possible existence of a quantum particle tunnelling phenomenon across EHs occurring through the stochastic belt of width δr. Hence, the minimum Lorentz γ-factor necessary to reach and eventually cross the tunneling region is calculated for each black hole mass interval. To this aim, the case of classical point particles with radial motion in the background of the Schwarzschild–deSitter metric is considered. It is proved that, in contrast with the purely classical case, the Lorentz factor required for the tunneling is not infinite, but acquires finite values scaling as $\gamma ∼M^{-1}$;
- To ascertain, from the conceptual point of view, whether the presence of a stochastic cosmological constant is sufficient to give all black hole event horizons a stochastic character.

In conclusion, the present theory proposes the possible existence of a new type of quantum tunneling phenomena that can arise in the surrounding of black hole event horizons. The tunneling mechanism is based purely on the stochastic quantum nature of the gravitational field predicted by CQG-theory. The important aspect to underline is that the transition region does not remain constant and/or of the order of the Planck length, and therefore independent of the physical properties of the background solution. This is a consequence of the stochastic mechanism pointed out here and based on CQG-theory. Instead, the resulting tunneling effect yields a background-dependent model which is affected by the black hole mass, which, in turn, generates the curved space–time itself.

## 2. Stochastic Quantum Gravity

In this section, we discuss the fundamental features of the manifestly covariant quantum gravity theory (CQG-theory) and its stochastic interpretation. We start recalling that a crucial feature of CQG-theory lies in the distinction between the quantum tensor $g_{\mu \nu }$, which identifies the continuum Lagrangian coordinates carrying the quantum physical properties of the gravitational field, and the background metric tensor ${\hat{g}}_{\mu \nu }$ which instead describes the geometry of space–time. By definition, the tensor $g_{\mu \nu }$ is such that $g^{\mu \nu }g_{\mu \nu }\neq \delta_{\mu }^{\mu }$, while, identically, the normalization condition ${\hat{g}}^{\mu \nu }{\hat{g}}_{\mu \nu }=4$ applies to the classical field. Accordingly, the quantum field $g_{\mu \nu }$ is allowed to exhibit a quantum dynamical behavior which deviates from ${\hat{g}}_{\mu \nu }$ and acquire a non-vanishing quantum momentum $\Pi_{\mu \nu }$.

The fundamental equation of CQG-theory is provided by the manifestly covariant 4−scalar quantum-gravity wave equation (CQG-wave equation) obtained in references [10,11]. This is parametrized in terms of an invariant proper-time parameter *s* defined with respect to the background metric tensor $\hat{g}$ as the proper-time associated with suitable classical sub-luminal geodesic trajectories, namely through the differential identity $ds^{2}={\hat{g}}_{\mu \nu }dr^{\mu }dr^{\nu }$ (see Ref. [10]). The CQG-wave equation takes the form of the hyperbolic first-order Eulerian PDE

(3) $$i\hbar \frac{d}{ds}\psi \left(s\right)=H_{R}^{\left(q\right)}\psi \left(s\right),$$

with *ℏ* being the reduced Planck constant, $\frac{d}{ds}={\left.\frac{d}{ds}\right|}_{s}+\frac{\partial }{\partial s}$ denoting the covariant s−derivative in Eulerian form, where the first differential operator is the customary covariant derivative evaluated at fixed *s* and the second one is a partial derivative acting on explicit proper-time dependences. Then, $H_{R}^{\left(q\right)}$ represents a suitable self-adjoint quantum Hamiltonian operator introduced in reference [10] and depending on the quantum momentum operator $\Pi_{\mu \nu }$. Furthermore, ψ(s) stands for $\psi \left(s\right)\equiv \psi \left(g,\hat{g},r,s\right)$ and denotes the 4−scalar quantum wave function associated with a graviton particle, which is defined for arbitrary *s* belonging to the time axis I≡R. Both explicit and implicit dependences on *s* are allowed, the latter enter through the 4−position vector $r\equiv r^{\mu }\left(s\right)$ of the background space–time. In addition, a functional dependence on both $g=\left\{g_{\mu \nu }\right\}$ and $\hat{g}=\left\{{\hat{g}}_{\mu \nu }\right\}$ are included, where $g_{\mu \nu }$ is the quantum generalized-coordinate field, which spans the 10−dimensional real vector space $U_{g}\subseteq R^{10}$ of the same wave-function, i.e., the set on which the associated quantum probability density function satisfying the Born rule, namely $\rho \left(s\right)={\left|\psi (s)\right|}^{2}$ (quantum PDF), is prescribed, while ${\hat{g}}_{\mu \nu }$ is the background metric tensor. We notice that the validity of the Born rule is strictly related to the manifestly-covariant nature of the relevant quantum-wave equation (in this case identified with Equation (3)) as well as the validity in the same context of the quantum unitarity principle.

The CQG-wave Equation (3) is equivalent to a set of quantum hydrodynamics equations obtained upon introducing an exponential representation for the complex field ψ(s), i.e., the so-called Madelung representation

(4) $$\psi \left(g,\hat{g},r,s\right)=\sqrt{\rho }\exp\left\{\frac{i}{\hbar }S^{\left(q\right)}\right\}.$$

The quantum fluid fields $\left\{\rho ,S^{\left(q\right)}\right\}\equiv \left\{\rho \left(g,\hat{g},r,s\right),S^{\left(q\right)}\left(g,\hat{g},r,s\right)\right\}$ identify, respectively, the 4−scalar quantum PDF and quantum phase-function. As a result, the same quantum fluid fields can be shown to satisfy a set of Bohmian equations, denoted as GR-quantum hydrodynamic equations (CQG-QHE). These are identified with the continuity and quantum Hamilton–Jacobi equations

(5) $$\begin{array}{ccc} \frac{d\rho }{ds}+\frac{\partial }{\partial g_{\mu \nu }}\left(\rho V_{\mu \nu }\right) & = & 0, \end{array}$$

(6) $$\begin{array}{ccc} \frac{dS^{\left(q\right)}}{ds}+H^{\left(q\right)} & = & 0, \end{array}$$

where $V_{\mu \nu }\equiv \frac{1}{\kappa }\frac{\partial S^{\left(q\right)}}{\partial g^{\mu \nu }}$ is the tensor “velocity” field, with κ being a dimensional constant which is related to the graviton mass estimate given in Ref. [10]. Equation (5), once integrated on the relevant configuration domain, implies the conservation of probability and hence validity of the said property of quantum unitarity. Furthermore, $H^{\left(q\right)}$ denotes the effective quantum Hamiltonian density

(7) $$H^{\left(q\right)}=\frac{1}{2\kappa }\frac{\partial S^{\left(q\right)}}{\partial g^{\mu \nu }}\frac{\partial S^{\left(q\right)}}{\partial g_{\mu \nu }}+V_{QM}+V_{o}+V_{F},$$

where $V_{o}$ and $V_{QM}$ identify, respectively, the vacuum effective potential and quantum Bohm interaction potential [35]. They are given by

(8) $$V_{o}=\kappa \left(2-\frac{1}{4}g^{\mu \nu }g_{\mu \nu }\right)g^{\alpha \beta }{\hat{R}}_{\alpha \beta },$$

(9) $$V_{QM}\equiv \frac{\hbar^{2}}{8\kappa }\frac{\partial \ln\rho }{\partial g^{\mu \nu }}\frac{\partial \ln\rho }{\partial g_{\mu \nu }}-\frac{\hbar^{2}}{4\kappa }\frac{\partial^{2}\rho }{\rho \partial g_{\mu \nu }\partial g^{\mu \nu }},$$

where ${\hat{R}}_{\alpha \beta }$ is the Ricci tensor evaluated in terms of the background metric tensor ${\hat{g}}_{\alpha \beta }$. In addition, $V_{F}$ is the potential of external sources. In the case of the Schwarzschild–deSitter space–time, this is associated with the central mass *M* generating the singular metric of the Schwarzschild black hole.

We remark that Equation (6) can be proved to be equivalent to a set of 4−tensor quantum Hamilton equations. They are intended as quantum hydrodynamic equations associated with the quantum wave function ψ, and prescribed in terms of the Hamiltonian hydrodynamic state $x\equiv (g_{\mu \nu },\Pi^{\mu \nu })$, with $\Pi^{\mu \nu }=\frac{\partial S^{\left(q\right)}}{\partial g_{\mu \nu }}$. Hence, the set $\left\{x,H^{\left(q\right)}\right\}$ defines a quantum Hamiltonian structure. The resulting manifestly covariant quantum Hamilton equations take the form of evolution equations in terms of the proper-time invariant parameter *s* and are written as

(10) $$\begin{array}{ccc} \frac{d}{ds}g^{\mu \nu } & = & \frac{\Pi^{\mu \nu }}{\alpha L}, \end{array}$$

(11) $$\begin{array}{ccc} \frac{d}{ds}\Pi_{\mu \nu } & = & -\frac{\partial }{\partial g^{\mu \nu }}\left(V_{o}+V_{QM}+V_{F}\right). \end{array}$$

These are subject to generic initial conditions of the type $x\left(s_{o}\right)=x_{o}\equiv \left(g_{\left(o\right)}^{\mu \nu }\equiv g^{\mu \nu }\left(s_{o}\right),\Pi_{\left(o\right)\mu \nu }\equiv \Pi_{\mu \nu }\left(s_{o}\right)\right)$.

The fluid representation of CQG-theory cen be cast in terms of a trajectory-based formulation. In the case of CQG-theory, this is provided by the Generalized Lagrangian Path theory (GLP-theory) developed in reference [36]. In summary, this is achieved in terms of a generalized Lagrangian-path (GLP) representation for the quantum Lagrangian field $g_{\mu \nu }\left(s\right)$ of the form

(12) $$g_{\mu \nu }\left(s\right)=\Delta g_{\mu \nu }\left(\alpha \right)+G_{\mu \nu }\left(s\right),$$

where $G_{\mu \nu }\left(s\right)$ denotes a suitably prescribed reference s−dependent quantum field. This is associated with a Lagrangian Path $\left\{G_{\mu \nu }\left(s\right),s\in I\right\}$, which can effectively be treated as deterministic if its initial value $G_{\mu \nu }\left(s_{o}\right)$ is considered deterministic. Instead, the tensor displacement field $\Delta g_{\mu \nu }\left(\alpha \right)$ is assumed as an arbitrary symmetric and s−independent stochastic tensor field. It describes the stochastic fluctuations in the quantum field tensor $g_{\mu \nu }$ with a given probability density $\rho_{\alpha }\left(s\right)$. The stochastic character of CQG-theory in this representation emerges, therefore, as a natural consequence of Equation (12) and is associated with $\Delta g_{\mu \nu }\left(\alpha \right)$. The meaning of Equation (12) is that, for each (deterministic) Lagrangian trajectory $\left\{G_{\mu \nu }\left(s\right),s\in I\right\},$ there are infinite stochastic GLP’s $\left\{g_{\mu \nu }\left(s\right),s\in I\right\}$. In particular, the prescription of the tensor displacement field $\Delta g_{\mu \nu }\left(\alpha \right)$ is taken as having a dependence of the form

(13) $$\Delta g_{\mu \nu }\left(\alpha \right)=\Delta g_{\mu \nu }\left(\alpha \right)\left(\Delta g_{\mu \nu },\alpha ,{\hat{g}}_{\mu \nu }\right).$$

Here, $\alpha \in \left[\alpha_{0},\alpha_{1}\right]\subseteq R$,with $\alpha_{0}<\alpha_{1}$, is a real 4−scalar stochastic parameter independent of $g_{\mu \nu }$ and *s* and with bounded support, while $\Delta g_{\mu \nu }$ is a stochastic tensor independent of α and subject to the condition $\frac{d}{ds}\Delta g_{\mu \nu }=0$.

Based on these preliminaries, one can prove that the solution to the continuity equation takes the form $\rho_{\alpha }\left(s\right)\equiv \rho \left(\Delta g\left(\alpha \right),\alpha ,\hat{g}\left(s,\alpha \right)\right)$, where

(14) $$\rho_{\alpha }\left(s\right)=\rho_{G}\left(s,\alpha \right)\exp\left\{-\int_{s_{o}}^{s}ds^{\prime }\frac{\partial V_{\nu }^{\mu }\left(s^{\prime }\right)}{\partial g_{\nu }^{\mu }\left(s^{\prime }\right)}\right\}.$$

The tensor “velocity field” $V_{\nu }^{\mu }\left(s\right)$ is assumed to be independent of α, and $\rho_{G}\left(s,\alpha \right)$ is the shifted Gaussian PDF (denoted as α−Gaussian PDF) which is expressed in terms of the stochastic tensor displacement $\Delta g_{\mu \nu }\left(\alpha \right)$ as

(15) $$\rho_{G}\left(s,\alpha \right)=K\exp\left\{-\frac{{\left(\Delta g\left(\alpha \right)-\hat{g}\left(s,\alpha \right)\right)}^{2}}{r_{th}^{2}}\right\}g\left(\alpha ,\varepsilon \right),$$

with g(α,ε) denoting an in principle arbitrary 4−scalar stochastic PDF. In the following, for definiteness g(α,ε) is identified with the Gaussian PDF

(16) $$g\left(\alpha ,\varepsilon \right)=N\exp\left\{-\frac{\alpha^{2}}{\varepsilon^{2}}\right\},$$

with ordinarily finite support, so that $\alpha \in \left[\alpha_{0},\alpha_{1}\right]\subset R$, while *N* is a normalization constant and ε is a suitably small dimensionless factor to be assumed ε≪1. In Equation (15), *K* is, therefore, the normalization factor

(17) $$K={\left[\int_{U_{g}}d\left(\Delta g\right)\int_{\alpha_{0}}^{\alpha_{1}}d\alpha \exp\left\{-\frac{{\left(\Delta g\left(\alpha \right)-\hat{g}\left(s,\alpha \right)\right)}^{2}}{r_{th}^{2}}\right\}g\left(\alpha ,\varepsilon \right)\right]}^{-1}.$$

In Equation (15) the tensors $\Delta g\left(\alpha \right)\equiv \Delta g_{\mu \nu }\left(\alpha \right)$ and $\hat{g}\left(s,\alpha \right)\equiv {\hat{g}}_{\mu \nu }\left(s,\alpha \right)$ identify the generalized displacement tensor $\Delta g_{\mu \nu }$ of GLP theory and the background metric tensor. Both are now admitted to generally depend on α itself, while $\hat{g}$ can also depend on the proper-time *s*. Similarly, in the same equation, the exponent ${\left(\Delta g\left(\alpha \right)-\hat{g}\left(s,\alpha \right)\right)}^{2}$ stands for the 4−scalar defined as ${\left(\Delta g\left(\alpha \right)-\hat{g}\left(s,\alpha \right)\right)}^{2}\equiv \left(\Delta g_{\mu \nu }\left(\alpha \right)-{\hat{g}}_{\mu \nu }\left(s,\alpha \right)\right)\left(\Delta g^{\mu \nu }\left(\alpha \right)-{\hat{g}}^{\mu \nu }\left(s,\alpha \right)\right)$.

The solution $\rho_{\alpha }\left(s\right)$ in Equation (15) represents a probability density which is consistent with the unitarity principle, so that, by construction

(18) $$\langle 1\rangle =\int_{U_{g}}d\left(\Delta g\right)\int_{\alpha_{0}}^{\alpha_{1}}d\alpha \rho_{\alpha }\left(s\right)=1.$$

This means that the quantum continuity Equation (5) preserves quantum unitarity. In addition, it yields the second-type emergent-gravity feature of CQG-theory (see reference [36]). Accordingly, the average expectation value of the stochastic tensor $\Delta g_{\mu \nu }\left(\alpha \right)$ coincides with the background metric tensor, namely

(19) $$\langle \Delta g_{\mu \nu }\left(\alpha \right)\rangle =\int_{U_{g}}d\left(\Delta g\right)\int_{\alpha_{0}}^{\alpha_{1}}d\alpha \left[\Delta g_{\mu \nu }\left(\alpha \right)\rho_{\alpha }\left(s\right)\right]={\hat{g}}_{\mu \nu }.$$

Conversely, since the distribution of α is a Gaussian centered around zero and with width ε, its stochastic average is identically null, namely

(20) $$\langle \alpha \rangle =\int_{U_{g}}d\left(\Delta g\right)\int_{\alpha_{0}}^{\alpha_{1}}d\alpha \left[\alpha \rho_{\alpha }\left(s\right)\right]=0.$$

We further notice that different realizations of Equation (14) could be distinguished, depending on the functional form of the half-width parameter ε, according to the discussion reported in reference [31]. However, for the scope of the present work and without limitations, in the following, we shall adopt the framework corresponding to assuming ε=const.≪1 everywhere. This choice preserves the validity of the quantum continuity equation and is, therefore, consistent with the unitary principle and, correspondingly, of a quantum-unitary formulation of CQG-theory. In this picture, Equation (15) preserves its validity as a solution to the continuity equation, and the stochastic PDF (14) holds in the whole space–time.

## 3. Stochastic Quantum-Modified Einstein Equations

In this Section, we detail the derivation of the Einstein field equations from the stochastic formulation of CQG-theory and related quantum Hamilton equations. This follows without performing the semiclassical limit ℏ→0, by invoking Equations (10) and (11) and imposing to the initial Hamiltonian state $x\left(s_{o}\right)$ “equilibrium” initial conditions of the type

(21) $$x\left(s_{o}\right)=\left(g_{\left(o\right)}^{\mu \nu }\equiv g^{\mu \nu }\left(s_{o}\right),\Pi_{\left(o\right)\mu \nu }\equiv 0\right).$$

In this way, the initial quantum tensor $g^{\mu \nu }$ coincides with the background one and its corresponding momentum (i.e., essentially its covariant derivative) identically vanishes. As a result, Equations (10) and (11) reduce to the single equation

(22) $${\left.\frac{\partial }{\partial g^{\mu \nu }}\left(V_{o}+V_{QM}+V_{F}\right)\right|}_{g_{\mu \nu }={\hat{g}}_{\mu \nu }}=0,$$

which contains the information by the Hamiltonian potential.

Then, invoking the definitions (8) and (9) for the potential terms $V_{o}$ and $V_{QM}$, respectively, it is straightforward to perform the differentiation with respect to $g^{\mu \nu }$ and evaluate the result for $g_{\mu \nu }={\hat{g}}_{\mu \nu }$ according to Equation (21). A similar calculation can be performed on the external potential $V_{F}$. This is assumed to be assigned and to correspond here to the central point mass *M* located at the origin of the reference system, as is customary in the well-known derivation of the classical Schwarzschild solution. This yields the following form for the resulting Einstein field equations

(23) $${\hat{R}}_{\mu \nu }-\frac{1}{2}\hat{R}{\hat{g}}_{\mu \nu }\left(s,\alpha \right)=T_{\mu \nu }^{\left(c\right)}+B_{\mu \nu }^{\left(\alpha \right)},$$

where $T_{\mu \nu }^{\left(c\right)}$ identifies the stress-energy tensor generated by classical sources (i.e., in the present case, the central point mass). Instead, the symmetric tensor $B_{\mu \nu }^{\left(\alpha \right)}$ carries the α−stochastic quantum contribution arising from the Bohm potential. In the following, Equation (23) will be referred to as α−*stochastic quantum-modified EFE*. In particular, the stochastic tensor $B_{\mu \nu }^{\left(\alpha \right)}$ is obtained as

(24) $$B_{\mu \nu }^{\left(\alpha \right)}\equiv {\left.-\frac{1}{\kappa }\frac{\partial }{\partial g^{\mu \nu }}V_{QM}\right|}_{g_{\mu \nu }={\hat{g}}_{\mu \nu }}=\frac{\hbar^{2}}{\kappa^{2}}\frac{f(s)}{r_{th}^{4}}\left(\Delta g_{\mu \nu }\left(\alpha \right)-{\hat{g}}_{\mu \nu }\left(s,\alpha \right)\right),$$

where f(s) is a suitably prescribed 4−scalar function depending on proper-time *s* and satisfying a given differential equation determined in Ref. [32]. Its value for the initial condition $s=s_{o}$ is such that $f(s_{o})=1$. We define the proper-time-dependent cosmological constant $\Lambda_{CQG}\left(s\right)$ arising due to the Bohm quantum vacuum interaction among massive gravitons as

(25) $$\Lambda_{CQG}\left(s\right)=\frac{\hbar^{2}}{\kappa^{2}}\frac{f(s)}{r_{th}^{4}}=\Lambda_{CQG}\left(s_{o}\right)f\left(s\right),$$

where

(26) $$\Lambda_{CQG}\left(s_{o}\right)=\frac{\hbar^{2}}{\kappa^{2}}\frac{1}{r_{th}^{4}}$$

is its constant initial value. Then, we can write Equation (23) in the explicit form

(27) $${\hat{R}}_{\mu \nu }-\frac{1}{2}\hat{R}{\hat{g}}_{\mu \nu }\left(s,\alpha \right)=T_{\mu \nu }^{\left(c\right)}-\Lambda_{CQG}\left(s\right){\hat{g}}_{\mu \nu }\left(s,\alpha \right)+\Lambda_{CQG}\left(s\right)\Delta g_{\mu \nu }\left(\alpha \right),$$

where, on the rhs, the last tensor carries the stochastic contributions due to $\Delta g_{\mu \nu }$ and α. This function carries the proper-time dependence of the quantum-gravity cosmological constant, which is, in this way, transferred to the metric tensor ${\hat{g}}_{\mu \nu }$.

Following the prescription reported in reference [31], we can introduce a convenient representation for the tensor $\Delta g_{\mu \nu }\left(\alpha \right)$ which is also consistent with the constraints set by the underlying quantum GLP theory by letting

(28) $$\Delta g_{\mu \nu }\left(\alpha \right)=\Delta g_{\mu \nu }+\alpha {\hat{g}}_{\mu \nu }\left(s,\alpha \right),$$

in which the two contributions due to $\Delta g_{\mu \nu }$ and α are decoupled. This shows that $\Delta g_{\mu \nu }$ is the tensorial term associated with the stochastic GLP fluid trajectories, while the term proportional to α plays an analogous role to a stochastic pressure. Based on this representation, we can now further elaborate the tensor Equation (27) following the procedure established in reference [31]. Thus, in Equation (27), we first impose the *deterministic LP-limit* $\Delta g_{\mu \nu }\to 0$, corresponding to the collapse of the stochastic quantum GLP trajectories on the unique LP trajectory. On the other hand, we retain the 4−scalar “pressure” contribution $\alpha {\hat{g}}_{\mu \nu }$, which is, therefore, required not to vanish when the LP-limit is imposed. In this way, we have singled out the 4−scalar α−stochastic contribution carried by $\Delta g_{\mu \nu }\left(\alpha \right)$. This is not related to the GLP/LP parametrization of the quantum wave equation like $\Delta g_{\mu \nu }$ is, but is associated with an intrinsic stochastic effect admitted by the quantum PDF solution of the continuity equation and, therefore, intrinsic to CQG-theory. Finally, collection of these requirements yields the following representation for the α−stochastic quantum-modified EFE

(29) $${\hat{R}}_{\mu \nu }-\frac{1}{2}\hat{R}{\hat{g}}_{\mu \nu }\left(s,\alpha \right)=T_{\mu \nu }^{\left(c\right)}-\Lambda_{CQG}\left(s\right)\left(1-\alpha \right){\hat{g}}_{\mu \nu }\left(s,\alpha \right),$$

in which the stochastic term is made explicit through the 4−scalar parameter α. This is the form of EFE that will be adopted below to determine a corresponding stochastic space–time metric tensor in the Schwarzschild–deSitter geometry and the subsequent study of stochastic effects across the black-hole event horizon.

## 4. Stochastic Schwarzschild–deSitter Background Solution

The virtue of the representations (28) and (29) is that the dependence on the stochastic parameter α remains linearly proportional to the CQG expression of the cosmological constant and to the space–time metric tensor ${\hat{g}}_{\mu \nu }$. If we identify the classical stress-energy tensor $T_{\mu \nu }^{\left(c\right)}$ as being due to the central point mass *M* located at the origin of the coordinate system $r^{\mu }=r_{o}^{\mu }$, then we can see that Equation (29) remains formally analogous to the classical EFE generating the Schwarzschild–deSitter solution. The only difference is that here, the CC Λ is replaced with the CQG 4−scalar $\Lambda_{CQG}\left(s\right)$ times the stochastic factor $\left(1-\alpha \right)$. In the following, we denote for brevity $\Lambda \equiv \Lambda_{CQG}\left(s\right)$, so that, as a consequence, one can identify the stochastic quantum-modified CC $\Lambda_{\left(\alpha \right)}$ carrying the stochastic contribution according to the following prescription

(30) $$\Lambda_{\left(\alpha \right)}\equiv \Lambda \left(1-\alpha \right).$$

In order to warrant that $\Lambda_{\left(\alpha \right)}>0$ and treating the parameter α as a quantum correction, it is required that $\alpha \in \left[\alpha_{0},\alpha_{1}\right]\ll 1$, which is assumed to be characterized by the PDF given by Equation (16). This is the only contribution that carries the stochastic effect generated by quantum-gravity on the background metric tensor. The latter is, therefore, correspondingly replaced with

(31) $${\hat{g}}_{\mu \nu }\to {\hat{g}}_{\mu \nu }\left(s,\alpha \right),$$

where the explicit dependence on both the proper time and the stochastic parameter α induced by $\Lambda_{\left(\alpha \right)}$ are indicated. It follows that the stochastic Equation (29) necessarily admits a corresponding *stochastic Schwarzschild–deSitter solution* depending on the cosmological constant Λ and the parameter α.

In particular, upon introducing generalized spherical coordinates (ct,r,ϑ,φ), assuming spherical symmetry, the background metric tensor can be written as ${\hat{g}}_{\mu \nu }=diag\left\{B,B^{-1},r^{2},r^{2}\sin^{2}\vartheta \right\}$. The corresponding Riemann distance takes the form $ds^{2}=Bc^{2}dt^{2}-B^{-1}dr^{2}+r^{2}d\Omega^{2}$. Here, the 4−scalar function *B* is given by

(32) $$B\equiv 1-\frac{R_{s}}{r}-\frac{r^{2}}{A_{\alpha }^{2}},$$

so that the line element is written explicitly as

(33) $$ds^{2}=\left(1-\frac{R_{s}}{r}-\frac{r^{2}}{A_{\alpha }^{2}}\right)c^{2}dt^{2}-\frac{1}{\left(1-\frac{R_{s}}{r}-\frac{r^{2}}{A_{\alpha }^{2}}\right)}dr^{2}+r^{2}d\Omega^{2}.$$

The Schwarzschild radius $R_{s}$ and the stochastic deSitter parameter $A_{\alpha }^{2}$ are defined, respectively, as

(34) $$\begin{array}{ccc} R_{s} & \equiv & \frac{2GM}{c^{2}}, \end{array}$$

(35) $$\begin{array}{ccc} A_{\alpha }^{2} & \equiv & \frac{3}{\Lambda_{\left(\alpha \right)}}=\frac{3}{\Lambda \left(1-\alpha \right)}. \end{array}$$

The Schwarzschild–deSitter space–time has two event horizons (EH), respectively, the one analogous to the Schwarzschild metric and generated by the central BH, to be denoted $r_{EH}$, and the cosmological one analogous to the deSitter EH, to be denoted $r_{dS}$. The location of the two EHs is modified with respect to the pure Schwarzschild or deSitter separate cases, because of the presence of the combined non-linear effect of the CC $\Lambda_{\left(\alpha \right)}$ and the central mass *M*. In spherical symmetry, the two EHs are represented by spherical surfaces at different radii, so that their location can be obtained by solving the algebraic equation

(36) $$g_{00}=0.$$

Invoking the line element (33) implies searching for the roots of the third-order equation

(37) $$1-\frac{R_{s}}{r}-\frac{r^{2}}{A_{\alpha }^{2}}=0.$$

Only two roots have physical meaning, and they are expressed as (see [37,38])

(38) $$r_{\pm }=\frac{2}{\sqrt{\Lambda_{\left(\alpha \right)}}}\cos\left[\frac{\pi }{3}\pm \frac{1}{3}\arccos\left(\frac{3R_{s}}{2}\sqrt{\Lambda_{\left(\alpha \right)}}\right)\right],$$

where, respectively, $r_{+}\equiv r_{EH}$ and $r_{-}\equiv r_{dS}$. In the following subsections, we determine asymptotic estimates for the two roots separately.

### 4.1. Black-Hole EH Asymptotic Estimate

Starting from the exact solution (38), we consider here the root $r_{+}\equiv r_{EH}$ identifying the exact radial location of the black hole EH

(39) $$r_{EH}=\frac{2}{\sqrt{\Lambda_{\left(\alpha \right)}}}\cos\left[\frac{\pi }{3}+\frac{1}{3}\arccos\left(\frac{3R_{s}}{2}\sqrt{\Lambda_{\left(\alpha \right)}}\right)\right].$$

In this section, we develop an approximation scheme to obtain an asymptotic estimate of $r_{EH}$ suitable for later investigation of the new stochastic quantum-gravity contribution carried by the CC on the physical properties of the same event horizon. To this aim, we first introduce the notation

(40) $$\varphi \equiv \arccos\left(\frac{3R_{s}}{2}\sqrt{\Lambda_{\left(\alpha \right)}}\right),$$

so that $r_{EH}$ can be written in the compact form

(41) $$r_{EH}=\frac{2}{\sqrt{\Lambda_{\left(\alpha \right)}}}\cos\left[\frac{\pi }{3}+\frac{\varphi }{3}\right].$$

Expressing the cosine in terms of the sum of the two arguments yields

(42) $$r_{EH}=\frac{2}{\sqrt{\Lambda_{\left(\alpha \right)}}}\left[\cos\left(\frac{\pi }{3}\right)\cos\left(\frac{\varphi }{3}\right)-\sin\left(\frac{\pi }{3}\right)\sin\left(\frac{\varphi }{3}\right)\right],$$

where $\cos\left(\frac{\pi }{3}\right)=\frac{1}{2}$ and $\sin\left(\frac{\pi }{3}\right)=\frac{\sqrt{3}}{2}$. Substitution gives

(43) $$r_{EH}=\frac{1}{\sqrt{\Lambda_{\left(\alpha \right)}}}\left[\cos\left(\frac{\varphi }{3}\right)-\sqrt{3}\sin\left(\frac{\varphi }{3}\right)\right].$$

We then notice that, in Equation (40), the argument of arccos is a very small number, being proportional to $\sqrt{\Lambda_{\left(\alpha \right)}}$. Expanding in Taylor series the arccos function for small argument gives up to the first order in the expansion

(44) $$\frac{\varphi }{3}≃\frac{\pi }{6}-\frac{R_{s}}{2}\sqrt{\Lambda_{\left(\alpha \right)}}.$$

Then, representing the cosine and sine functions in Equation (43) in terms of the sum of the arguments yields

(45) $$r_{EH}=\frac{2}{\sqrt{\Lambda_{\left(\alpha \right)}}}\sin\left(\frac{R_{s}}{2}\sqrt{\Lambda_{\left(\alpha \right)}}\right).$$

We now use the Taylor expansion for sine function. Retaining up to third-order powers, so that $\sin\left(x\right)≃x-\frac{x^{3}}{6}$, then yields

(46) $$\sin\left(\frac{R_{s}}{2}\sqrt{\Lambda_{\left(\alpha \right)}}\right)≃\frac{R_{s}}{2}\sqrt{\Lambda_{\left(\alpha \right)}}-\frac{1}{6}{\left(\frac{R_{s}}{2}\sqrt{\Lambda_{\left(\alpha \right)}}\right)}^{3}.$$

Finally, inserting into Equation (45) gives

(47) $$r_{EH}≃\frac{2}{\sqrt{\Lambda_{\left(\alpha \right)}}}\left[\frac{R_{s}}{2}\sqrt{\Lambda_{\left(\alpha \right)}}-\frac{1}{6}{\left(\frac{R_{s}}{2}\sqrt{\Lambda_{\left(\alpha \right)}}\right)}^{3}\right]=R_{s}-\frac{R_{s}^{3}}{24}\Lambda_{\left(\alpha \right)}.$$

We have thus obtained, as a result, the final asymptotic estimate

(48) $$r_{EH}=R_{s}\left[1-\frac{R_{s}^{2}}{24}\Lambda_{\left(\alpha \right)}\right],$$

in which the contribution due to the stochastic CC enters as a first-order correction. We can see that the approximate solution recovers the correct value expected in the limit of vanishing CC, namely the exact Schwarzschild solution

(49) $$\lim_{\Lambda \to 0}r_{EH}=R_{s}.$$

In addition, when the stochastic CC corrections are retained, the magnitude of $r_{EH}$ is decreased with respect to the pure Schwarzschild case, as expected from the fact that the same CC generates an expansion (it effectively decreases the energy content due to the central mass, causing a decrement of the BH radius).

### 4.2. Cosmological EH Asymptotic Estimate

For completeness, here we also evaluate the modification of the cosmological deSitter EH in the case of Schwarzschild–deSitter spacetime. We consider, therefore, the root $r_{-}\equiv r_{dS}$ given by

(50) $$r_{dS}=\frac{2}{\sqrt{\Lambda_{\left(\alpha \right)}}}\cos\left[\frac{\pi }{3}-\frac{1}{3}\arccos\left(\frac{3R_{s}}{2}\sqrt{\Lambda_{\left(\alpha \right)}}\right)\right].$$

We proceed determining an appropriate asymptotic estimate for $r_{dS}$. To this aim, we first introduce again the notation in terms of φ given by Equation (40), so that $r_{dS}$ can be written in the compact form as

(51) $$r_{dS}=\frac{2}{\sqrt{\Lambda_{\left(\alpha \right)}}}\cos\left[\frac{\pi }{3}-\frac{\varphi }{3}\right].$$

Expressing the cosine in terms of the sum of the two arguments yields

(52) $$r_{dS}=\frac{1}{\sqrt{\Lambda_{\left(\alpha \right)}}}\left[\cos\left(\frac{\varphi }{3}\right)+\sqrt{3}\sin\left(\frac{\varphi }{3}\right)\right].$$

Representing, in Equation (52), the cosine and sine functions in terms of the sum of the arguments, and rearranging them after some algebra, one obtains

(53) $$r_{dS}=\frac{1}{\sqrt{\Lambda_{\left(\alpha \right)}}}\left[\sqrt{3}\cos\left(\frac{R_{s}}{2}\sqrt{\Lambda_{\left(\alpha \right)}}\right)-\sin\left(\frac{R_{s}}{2}\sqrt{\Lambda_{\left(\alpha \right)}}\right)\right].$$

We now use the Taylor expansion for sine and cosine functions for small arguments, so that $\cos\left(x\right)≃1-\frac{x^{2}}{2}$ and $\sin\left(x\right)≃x-\frac{x^{3}}{6}$. This gives respectively

(54) $$\cos\left(\frac{R_{s}}{2}\sqrt{\Lambda_{\left(\alpha \right)}}\right)≃1-\frac{1}{2}{\left(\frac{R_{s}}{2}\sqrt{\Lambda_{\left(\alpha \right)}}\right)}^{2},$$

(55) $$\sin\left(\frac{R_{s}}{2}\sqrt{\Lambda_{\left(\alpha \right)}}\right)≃\frac{R_{s}}{2}\sqrt{\Lambda_{\left(\alpha \right)}}-\frac{1}{6}{\left(\frac{R_{s}}{2}\sqrt{\Lambda_{\left(\alpha \right)}}\right)}^{3}.$$

Finally, inserting into Equation (53) and retaining corrections up to the second order gives

(56) $$r_{dS}=\sqrt{\frac{3}{\Lambda_{\left(\alpha \right)}}}\left[1-\frac{1}{\sqrt{3}}\frac{R_{s}}{2}\sqrt{\Lambda_{\left(\alpha \right)}}-\frac{1}{2}{\left(\frac{R_{s}}{2}\sqrt{\Lambda_{\left(\alpha \right)}}\right)}^{2}\right].$$

In this case, we can see that the approximate solution recovers the correct value expected in the limit of vanishing mass, or, equivalently, of vanishing $R_{s}$, namely

(57) $$\lim_{M\to 0}r_{dS}=\lim_{R_{s}\to 0}r_{dS}=\sqrt{\frac{3}{\Lambda_{\left(\alpha \right)}}},$$

which is the customary deSitter radius of the cosmological deSitter geometry with Λ≠0. In addition, in this case, the magnitude of the cosmological EH $r_{dS}$ is decreased by the correction due to the central BH, as expected from the fact that the same mass contrasts with the expansion of the CC and curves the universe.

## 5. Estimate of EH Belt Width

In this section, we show that the stochastic quantum-gravity corrections induced by the α parameter on the Schwarzschild–deSitter metric tensor imply a radical change in the structure of the black-hole EH. In fact, contrary to the purely classical solution, it is proven that, in the framework of the stochastic quantum-gravity and, more precisely, in the presence of a non-vanishing stochastic CC, the same EH is modified in a substantial way, so that it acquires a width. Thus, instead of representing a 2D surface (spherical surface), like in the classical case, the stochastic EH becomes now a region with prescribed width. This is determined by the same parameter α and its stochastic domain of support defined by the quantum PDF. This implies, in turn, that, due to stochastic quantum corrections, the quantum EH is no longer an impenetrable deterministic barrier with a fixed location and separating two “incommunicable” regions of space–time. Rather, it gives rise to a transition region which can permit, in principle, the quantum transition from inner and outer regions, i.e., particle tunneling phenomena. In the following, we shall denote this region as *EH belt*.

It must be stressed, however, that this quantity does not represent an invariant length, namely a 4−scalar. Instead, the EH belt width arises as a frame-dependent scale according to the choice of coordinate system. Therefore, the consequent feature to be mentioned is that the occurrence of the characteristic EH belt width has an independent character of the Planck length $l_{P}$. In particular, it has no connection with the concept of “minimum length”, usually adopted in the framework of the literature Generalized Uncertainty Principle theories [39,40,41] (and typically identified with $l_{P}$). The lack of an absolute minimal length is, in fact, a characteristic of CQG-theory. However, this feature does not prevent the possible existence of a characteristic physical EH belt width nor, as shown in reference [42], the existence of an effective characteristic standard deviation for the proper-time scale-length Δs (i.e., a 4−scalar characteristic scale length) which is associated with the corresponding (proper-time-conjugate canonical momentum) Heisenberg inequality.

We want to prove the existence of this effect on the EH, which is induced uniquely by the stochastic character of the quantum CC. In order to estimate the width of the EH belt, we start from the approximate analytical solution for $r_{EH}$ determined above by Equation (48). Invoking the definition of $\Lambda_{\left(\alpha \right)}$ from Equation (30), we can express the equation of $r_{EH}$, pointing out the explicit contribution of the stochastic parameter α, yielding

(58) $$r_{EH}=R_{s}\left[1-\frac{R_{s}^{2}}{24}\Lambda \left(1-\alpha \right)\right].$$

Given the Gaussian representation for the probability density of α, from the physical perspective, the condition ε≪1 means that the same distribution is very peaked, with a narrow half-width. Hence, one can reasonably assume that α varies randomly in the range $\alpha ∼\left[-\varepsilon ,+\varepsilon \right]$. This implies that the quantity $\left(1-\alpha \right)∼O\left(1\right)$ and remains positive.

The behavior of α implies the notable consequence that, similarly, the Schwarzschild–deSitter BH event horizon acquires a width, which represents a stochastic quantum belt. For an order-of-magnitude estimate, we can write $r_{EH}\in \left[r_{EH}^{\min},r_{EH}^{\max}\right]$, where spherical symmetry is assumed to hold. The evaluation of the extrema of such intervals can be estimated from Equation (58). Thus, assuming α∼ε≪1, to leading-order in ε one finds, respectively,

(59) $$\begin{array}{ccc} r_{EH}^{\min} & = & R_{s}\left[1-\frac{R_{s}^{2}}{24}\Lambda \left(1+\left|\varepsilon \right|\right)\right], \end{array}$$

(60) $$\begin{array}{ccc} r_{EH}^{\max} & = & R_{s}\left[1-\frac{R_{s}^{2}}{24}\Lambda \left(1-\left|\varepsilon \right|\right)\right]. \end{array}$$

To further elaborate the expression, we denote by

(61) $$r_{EH}^{SdS}=R_{s}\left[1-\frac{R_{s}^{2}}{24}\Lambda \right]$$

the value of the black hole EH of the classical “deterministic” Schwarzschild–deSitter metric, namely, its value in the absence of quantum-gravity-induced stochastic dependences. Therefore, the classical location $r_{EH}^{SdS}$ is subject to a maximum increase/decrease change given by the factor δr, namely

(62) $$r_{EH}=r_{EH}^{SdS}\pm \delta r,$$

where

(63) $$\delta r=\frac{R_{s}^{3}}{24}\Lambda \left|\varepsilon \right|$$

is the measure of the maximum width that the EH belt can acquire according to the present model. Inserting in the previous expression the definition of $R_{s}$ from Equation (34) yields

(64) $$\delta r=\frac{1}{24}{\left(\frac{2G}{c^{2}}\right)}^{3}\Lambda \left|\varepsilon \right|M^{3}.$$

From this, it is evident that δr scales as

(65) $$\delta r∼M^{3},$$

i.e., it is proportional to the cubic of the central black-hole mass *M*.

We can now estimate the magnitude of δr for relevant cases of astrophysical interest. The proportional constants in SI units are

$$\begin{array}{ccc} G & = & 6.67408\times 10^{-11}\;m^{3}{\mathrm{kg}}^{-1}s^{-2},\\ c & = & 3\times 10^{8}\;{\mathrm{ms}}^{-1},\\ \Lambda & = & 1.1056\times 10^{-52}\;m^{-2}, \end{array}$$

while the mass *M* is expressed in units of the solar mass as $M=\eta M_{⊙}$, where $M_{⊙}=2\times 10^{30}$ kg. It then follows that

(66) $$\delta r≃1.2\times 10^{-43}\left|\varepsilon \right|\eta^{3},$$

with δr being measured in meter. The value of the correction radius δr must then be compared with the Planck length $l_{p}≃1.6\times 10^{-35}$ m. For practical numerical estimates, we also set in the following $\varepsilon =10^{-2}$.

The following cases are then considered

(1) Stellar-mass black-holes with typical mass $M∼10M_{⊙}$. In this case we have η∼10, so that

(67) $$\delta r≃1.2\times 10^{-42},$$

and therefore $\delta r\ll l_{p}$.

(2) Intermediate-mass black-holes with typical masses in the range $M\in \left[10^{2}-10^{4}\right]M_{⊙}$. In this case $\eta =10^{2}-10^{4}$, and therefore

(68) $$\delta r≃1.2\times \left(10^{-39}-10^{-33}\right),$$

so that either $\delta r\ll l_{p}$ (higher bound) or $l_{p}≲\delta r$ (lower bound).

(3) Supermassive black-holes with typical masses in the range $M\in \left[10^{6}-10^{10}\right]M_{⊙}$. In this last example $\eta =10^{6}-10^{10}$, implying

(69) $$\delta r≃1.2\times \left(10^{-27}-10^{-15}\right),$$

which means that $\delta r\gg l_{p}$.

The following conclusions can be drawn:

- According to the present theoretical model based on the stochastic character of the quantum CC predicted by CQG-theory, the black-hole EH of the stochastic Schwarzschild–deSitter solution is identified with a transition region (between internal and external space-time domains) of width δr denoted EH belt, inside which the horizon is stochastically located. The typical radial size of the stochastic EH belt δr is not a constant. It differs generally from the Planck length $l_{P}$, which is customarily claimed as the characteristic size for the manifestation of black-hole quantum-gravity and tunneling phenomena. More precisely, δr is shown to depend parametrically on the central mass *M* of the black hole and, in particular, to scale as $\delta r∼M^{3}$;
- The numerical estimates given above of the radial width δr show that, in the case of supermassive black holes, this is typically orders of magnitude larger that the Planck length $l_{P}$. One finds instead that $l_{p}≲\delta r\ll l_{p}$ for intermediate-mass black holes, while for typical stellar-mass black holes, δr may drop well below $l_{P}$.

## 6. The Tunneling Lorentz Factor

In this section, we investigate the implications of the stochastic quantum-gravity solution obtained above and the prediction of the existence of the stochastic EH belt on the particle tunneling phenomenon. More precisely, we consider here the dynamics of classical particles, assumed to have a pure radial motion in the Schwarzschild–deSitter metric. In such a framework, we are interested in investigating the dependence of the Lorentz γ−factor, which is defined as

(70) $$\gamma \equiv \frac{1}{\sqrt{g_{00}}}.$$

Invoking Equation (33), we obtain the expression

(71) $$\gamma =\frac{1}{\sqrt{\left(1-\frac{R_{s}}{r}-\frac{r^{2}}{A_{\alpha }^{2}}\right)}},$$

which applies in the general stochastic picture.

On the other hand, in the particular case of the classical deterministic solution, the space–time metric tensor is characterized by a unique deterministic EH located at radial distance $r_{EH}{|}_{\alpha =0}$, namely, setting α=0 in the analytical solution for $r_{EH}$ given by Equation (39). If we evaluate the Lorentz γ-factor on such a classical EH, we obtain

(72) $${\left.\gamma \right|}_{r=r_{EH}{|}_{\alpha =0}}=\infty .$$

This expresses the characteristic feature of the classical EH of representing an impenetrable barrier. In such a framework, it is, therefore, meaningful to estimate the minimum Lorentz γ-factor required for classical particles to reach the domain associated with the EH belt in the stochastic quantum-gravity solution. There, one can expect particle tunneling phenomena to take place, to be induced by the same quantum nature of the gravitational field. For this reason, we refer to such a γ-factor as the *tunneling Lorentz factor*. Particles that possess this Lorentz factor are possible candidates to reach the stochastic EH belt and can take part in the new type of quantum-gravity tunneling mechanism generated by the stochastic character of the same EH due to the stochastic CC.

Let us, therefore, consider the stochastic Schwarzschild–deSitter quantum gravity solution and the properties of the corresponding black-hole EH belt. Assuming a small stochastic correction, the precise representation can be obtained by Taylor expansion of the exact analytical solution (39), namely, of the form

(73) $$r=r_{EH}{|}_{\alpha =0}+\alpha {\left.\frac{\partial r_{EH}}{\partial \alpha }\right|}_{\alpha =0}+O\left(\alpha^{2}\right).$$

The estimate of the linear α−correction can be identified with the EH width obtained in previous section. Thus, invoking Equation (62) and assuming that α varies randomly in the range $\alpha ∼\left[-\varepsilon ,+\varepsilon \right]$, we can take the maximum width amplitude. For the present calculation, we are interested in the “increased” solution and we can write

(74) $$r≃r_{EH}{|}_{\alpha =0}+\delta r,$$

where the absolute value of δr is given by Equation (63). Inserting Equation (74) into Equation (71), and approximating only in the correction terms $r_{EH}{|}_{\alpha =0}$ with $r_{EH}^{SdS}$ given by Equation (61), ignoring corrections of $O\left(R_{s}^{2}\Lambda \right)$ we obtain

(75) $$\gamma ≃\frac{1}{\sqrt{\frac{\delta r}{R_{s}}}}=\sqrt{\frac{R_{s}}{\delta r}}.$$

We notice that the same result can be equivalently obtained by introducing the asymptotic approximation

(76) $$\gamma =\frac{1}{\sqrt{\left(1-\frac{R_{s}}{\left(r_{EH}{|}_{\alpha =0}+\delta r\right)}-\frac{{\left(r_{EH}{|}_{\alpha =0}+\delta r\right)}^{2}}{A_{\alpha }^{2}}\right)}}≃\frac{1}{\sqrt{\left(1-\frac{R_{s}}{\left(R_{s}+\delta r\right)}\right)}}≃\frac{1}{\sqrt{\frac{\delta r}{R_{s}}}}.$$

In fact, one can start from the exact definition (71) using the expansion (74) and approximating $r_{EH}{|}_{\alpha =0}$ with the expression $r_{EH}^{SdS}$ given by Equation (61). Upon neglecting terms of $O\left(R_{s}^{4}\Lambda^{2}\right)$ and $O\left(R_{s}^{6}\Lambda^{3}\right)$, an explicit calculation brings the following expression

(77) $$\gamma ≃\frac{1}{\sqrt{\frac{R_{s}^{2}}{24}\Lambda \left|\varepsilon \right|}}.$$

Replacing the definition of $R_{s}$, we can obtain the explicit dependence of γ on the various parameters

(78) $$\gamma =\sqrt{\frac{24}{R_{s}^{2}\Lambda \left|\varepsilon \right|}}=\frac{c^{2}}{GM}\sqrt{\frac{6}{\Lambda \left|\varepsilon \right|}}=\frac{c^{2}\sqrt{6}}{G}\Lambda^{-1/2}{\left|\varepsilon \right|}^{-1/2}M^{-1}.$$

This shows that the γ-factor is inversely proportional to the mass of the BH, namely, it scales as $\gamma ∼M^{-1}$.

We can now estimate the numerical values of γ from Equation (78) for the three cases of black-hole mass intervals considered above, assuming again $\varepsilon =10^{-2}$

(1) Case of stellar-mass black-holes with $M∼10M_{⊙}$:

(79) $$\gamma ≃1.57\times 10^{23};$$

(2) Intermediate-mass black-holes with $M\in \left[10^{2}-10^{4}\right]M_{⊙}$:

(80) $$\gamma ≃1.57\times \left[10^{22}-10^{20}\right];$$

(3) Supermassive black-holes with $M\in \left[10^{6}-10^{10}\right]M_{⊙}$:

(81) $$\gamma ≃1.57\times \left[10^{18}-10^{14}\right].$$

We conclude that, contrary to the classical expectation that predicts the Lorentz factor to be infinite at the EH, the stochastic quantum gravity effect induced by the stochastic CC makes the Lorentz factor necessary to reach the stochastic black-hole EH belt finite. Particles able to enter the same stochastic belt are then candidates to generate particle tunneling phenomena across the EH. In fact, the EH is stochastic too, and therefore it cannot be located in a deterministic way at a fixed radial value. The fact that this tunneling effect is induced solely by the CC makes it very weak. The reason for this is that the stochastic behavior of the CC is maximum at the deSitter EH and, therefore, on cosmological scales, is then transferred to operate on local and much smaller black-hole scales of the order of $R_{s}$. Nevertheless, these calculations are enough to prove the possible existence of this phenomenon. This remains independent of other quantum-gravity mechanisms expected to operate at the Planck scale and predicted in the framework of other quantum gravity theories, like loop quantum gravity.

## 7. Conclusions

The theory of General Relativity is characterized by the occurrence of classical singularitites in the space–time metric tensor solution of the Einstein field equations. In the case of black-hole geometries, singularities can characterize either the black hole center or the event horizon. In classical GR the latter is identified with a deterministic two-dimensional surface which separates two neighboring, internal and external to the same surface, space-time subdomains. These are regarded as truly incommunicable regions. Although the nature of such singularities is different, it is generally believed that its appropriate treatment and eventual resolution (i.e., regularization) can only be reached by means of a quantum theory of the gravitational field.

In this paper, the subject of investigation is the quantum modifications of the classical black hole structure induced by stochastic quantum gravity. The focus refers, more precisely, to the physical properties of the quantum-modified black-hole event horizon. This has been accomplished in the framework of the stochastic formulation of the manifestly covariant quantum gravity theory. The latter predicts the existence of massive gravitons whose vacuum quantum interaction is described by the Bohm potential, which ultimately generates an exclusively quantum-produced stochastic cosmological constant in the Einstein field equations.

The main physical implication reached here is the expectation that actual black hole event horizons, in general, are not deterministic, due to the stochastic character of the quantum-produced cosmological constant. The result, which is an obvious implication of the theory developed here, has been investigated in detail in the case of the Scwarzschild–deSitter metric. According to the customary picture arising in classical GR, event horizons are represented by impenetrable two-dimensional surfaces of space–time. In contrast, we have shown that event horizons acquire a stochastic quantum character. This is inherited from the stochastic behavior of the quantum-produced cosmological constant. The basic implication is that, due the presence of such a kind of stochastic quantum effect, it is no longer possible to assign the event horizon a deterministic (i.e., unique) physical location in the space-time. The corresponding deterministic two-dimensional surface must be replaced by an effective, finite-size, stochastic domain. The notion of a classical event horizon surface must, therefore, be replaced by the new concept of stochastic event horizon belt. This realizes a transition region of frame-dependent radial width δr between internal and external subdomains, inside which the horizon has a certain probability of existence. The notable feature is that the probability distribution can be shown to be uniquely prescribed by quantum gravity theory itself, together with the related stochastic quantum-modified Einstein field equations.

An interesting physical implication of the theory developed here is that, based on estimates of the stochastic cosmological constant determined by CQG-theory, the amplitude of the frame-dependent radial size (δr) characteristic of the stochastic belt is not a model-independent quantity. Unlike most of the current phenomenological theories of quantum-gravity, it appears to be unrelated to the Planck length.

Instead, the typical width of the stochastic region surrounding the deterministic event horizon depends parametrically on the central mass *M* of the black hole, scaled as $\delta r∼M^{3}$. Thus, as a consequence, it is found that, in the case of supermassive black holes, with mass in the range $M\in \left[10^{6}-10^{10}\right]M_{⊙}$, typically δr, can be estimated to be orders of magnitude larger than the Planck length $l_{P}$. Then, for intermediate-mass black holes with mass in the range $M\in \left[10^{2}-10^{4}\right]M_{⊙}$, it is found that $l_{p}≲\delta r\ll l_{p}$. Finally, for typical stellar-mass black holes with mass $M∼10M_{⊙}$, δr may drop well below $l_{P}$. From the physical point of view, the existence of the stochastic event horizon belt has been interpreted as supporting the onset of particle tunneling phenomena across the same horizon. Then, as a related result, we calculated the minimum Lorentz γ-factor required for classical particles with a radial motion in the Schwarzschild–deSitter metric to reach the stochastic belt and, therefore, to enter the space–time domain where the tunneling effect has a non-vanishing probability to occur. Contrary to the classical case which predicts the Lorentz factor to be infinite on the horizon, in the quantum stochastic framework considered here, the same Lorentz factor has a finite value. It depends on the mass of the central black-hole and scales as $\gamma ∼M^{-1}$.

The conclusions drawn in this work provide new insight into the quantum properties of black holes and might have crucial implications for the physics of quantum tunneling phenomena that are expected to arise across stochastic event horizons. The conceptual implications of the solution considered in the paper are challenging and provide a new quantum-gravity mechanism for the treatment of black-hole event horizons. In fact, it has been shown that these can exhibit a quantum stochastic nature. This is ultimately induced by the ubiquitous existence of a quantum cosmological constant predicted by the manifestly covariant quantum gravity theory and arising from the fundamental nonlinear Bohm interaction among massive gravitons. The novel picture discussed in the paper provides a physically meaningful framework for the possible generation and emission of massive gravitons, particle or fields across black-hole subdomains. The conclusions reached here are, therefore, meaningful in the context of quantum gravity, theoretical astrophysics and cosmology. Their potential physical relevance concerns the characterization of quantum phenomena and tunnelling effects that can occur in the surroundings of event horizons.

## Data Availability

Not applicable.
